# Genetic diversity and population structure of wild and cultivated *Camellia tetracocca* Chang assessed by ILP markers: insights for conservation of an endangered tea species

**DOI:** 10.1186/s12870-026-08815-0

**Published:** 2026-04-23

**Authors:** Ziyun Yang, Manyun Zhao, Shukui Chang, Wenmin Guo, Yafei Cui, Di Meng, Lifei Wang, Lingling Zheng, Huizhen Hu, Xiaoxia Huang, Xiaohan Xu, Xia Li, Xiaomao Cheng

**Affiliations:** 1 Yunnan Key Laboratory of Landscape Plant Resource Cultivation and Application, College of Landscape Architecture and Horticulture Sciences, Southwest Forestry University, Kunming, 650224 China; 2 Biotechnology and Germplasm Resources Institute, Yunnan Academy of Agricultural Sciences, Kunming, 650205 China; 3 Pu’an County Tea Development Center, Puan, 561599 China; 4 Southwest Survey and Planning Institute, National Forestry and Grassland Administration, Kunming, 650031 China; 5 Faculty of Foreign Languages (International College), Southwest Forestry University, Kunming, 650224 China

**Keywords:** *Camellia tetracocca* Chang, ILP marker, genetic diversity, population structure, core collection

## Abstract

**Background:**

Qingshan Town, Guizhou Province, represents the core distribution area of wild *Camellia tetracocca* Chang and a key production area of ‘Pu’an hong’ tea. This region has a long history of tea cultivation and harbors rich tea germplasm resources. As an endemic and endangered tea species in Guizhou, *C. tetracocca* Chang is threatened by habitat fragmentation and genetic diversity loss, highlighting the urgent need for systematic genetic analysis and conservation research. To facilitate the conservation and utilization of tea germplasm in Qingshan Town, 192 tea accessions representing four populations (wild ancient tea plant Ⅰ, wild ancient tea plant Ⅱ, cultivated ancient tea plant, and cultivated tea plant) were analyzed for genetic diversity using intron length polymorphism (ILP) markers.

**Results:**

A total of 180 alleles were detected across 40 ILP loci, with a mean observed alleles (*Na*) of 4.45. The average observed heterozygosity (*H*_o_) was 0.22, expected heterozygosity (*H*_e_) 0.59, and Nei’s gene diversity (*H*) 0.59. The mean Shannon’s information index (*I*) was 1.11, and the mean polymorphic information content (*PIC*) was 0.53. Structure analysis revealed that the 192 individuals could be divided into three subpopulations, which was consistent with the results of UPGMA clustering and principal coordinate analysis (PCoA). Furthermore, a core germplasm collection comprising of 29 accessions was successfully established.

**Conclusion:**

Our results indicate that the tea germplasm in Qingshan Town maintains high genetic diversity, and the constructed core germplasm collection effectively represents the genetic diversity of the whole collection. This study provides a theoretical foundation for breeding elite tea cultivars and conserving the genetic resources of this endangered tea species.

**Supplementary Information:**

The online version contains supplementary material available at 10.1186/s12870-026-08815-0.

## Introduction

 Tea plants are among the world’s most economically important beverage crops, valued for their abundant secondary metabolites that contribute to their unique flavor and numerous health-promoting properties [1, 2]. Native to the Yunnan–Guizhou Plateau in China, tea plants have been cultivated for more than 3,000 years, with their cultivation range gradually expanding to eastern, southeastern, and southern China over millennia [3, 4]. As a self-incompatible species, tea plants exhibit high heterozygosity and extensive genetic diversity, shaped by long-term natural selection and artificial domestication [5]. Local tea varieties differ significantly in morphological characteristics, processing adaptability, cold tolerance, and yield potential. Investigating the genetic diversity of these diverse tea germplasm resources is critical for clarifying the origin and evolutionary history of both wild and domesticated tea varieties [6], and it also provides a solid foundation for exploring gene functions and facilitating targeted plant breeding programs. This inherent genetic diversity is essential for enhancing adaptability to diverse environmental conditions and developing new varieties with improved agronomic and quality traits.

Genetic variation in tea plants is shaped by multiple factors, including domestication, geographic isolation, seed propagation, selective breeding, and hybridization [7, 8]. This variation is vital for tea plant adaptation to different environments and the development of new varieties with desirable traits. Pu’an County has a warm, humid climate with optimal temperatures and abundant precipitation, providing favorable conditions for tea growth. Due to its topographical conditions, wild *C. tetracocca* is relatively scattered, primarily distributed on slopes in Majiaping Village, Pubai Forest Farm, and other mountainous forested areas at elevations ranging from 1700 to 1950 m a.s.l. in southwest Guizhou Province [9]. This unique geographical environment endows the tea germplasm with strong regional specificity, limiting its survival after transplantation. Currently, *C. tetracocca* exists in wild, cultivated, and terraced types, each with distinct characteristics influenced by their growing environments [10]. Under this unique geographical setting, *C. tetracocca* has evolved distinct genetic and chemical profiles, making it a valuable resource for breeding new tea cultivars with improved flavor and enhanced health-promoting properties [11]. The presence of specific secondary metabolites, including catechins and theacrine, provides opportunities for breeding tea plants with superior antioxidant capacity and reduced caffeine content, aligning with the increasing consumer demand for healthier tea products [12]. However, the tea germplasm in Qingshan Town remains insufficiently characterized due to a lack of comprehensive genetic studies.

A core collection is defined as a small subset of germplasm resources carefully selected to represent the maximum genetic variation of the entire germplasm bank [13]. As perennial woody plants, tea plants exhibit self-incompatibility, high genetic heterozygosity, and are widely distributed. Therefore, constructing a core collection that maximally captures the genetic diversity of the original germplasm has become a key strategy for the efficient conservation and precise utilization of tea germplasm resources. In recent years, extensive research on core collections has been conducted in various crops, providing valuable theoretical and technical insights for the integration and utilization of tea germplasm resources. For example, Fu et al. [14] constructed a representative core collection of *Populus alba*; Todd et al. [15] established a breeding foundation set focused on genetic diversity by evaluating global cacao (*Theobroma cacao*) resources, including accessions with high productivity and pathogen resistance. Huo et al. [16] used a core collection to accelerate the discovery of the anthocyanin biosynthesis regulator (ANS) in *Lactuca sativa* and successfully developed a new high-anthocyanin lettuce variety.

In China, research on the construction of core collections for tea germplasm started early and has been gradually deepened. In 2011, Wang et al. [17] established the national-scale tea core collection, selecting approximately 19.8% of accessions from more than 2,000 entries in the National Tea Germplasm Repository. Subsequently, a series of regional core collections of local tea germplasm have been developed. For instance, Ruan et al. [18] constructed a core collection for Huangshan tea germplasm based on the genetic diversity assessment of local landraces in the Huangshan area of Anhui Province; Wang et al. [19] (2025) focused on the characteristic tea resources in the Lu’an area and established a core collection with strong local adaptability; Liu et al. [20] analyzed landrace tea populations in the Rucheng area and constructed a core collection representing local genetic diversity; and Niu et al. [21] established a local core collection covering typical ecological types across Guizhou Province based on the systematic evaluation of regional tea germplasm resources. The establishment of local tea core collections not only significantly reduces conservation costs and optimizes germplasm resources but also improves the efficiency of germplasm identification and regeneration. In addition, such core collections provide a high-quality genetic material platform for the discovery of elite genes specific to local tea resources and the design of targeted cross combinations in tea breeding.

Molecular marker technology is a powerful tool for constructing plant core germplasm. Over the past two decades, various molecular marker techniques have been developed and widely used in diverse plant systems. Previous studies have employed random amplified polymorphic DNA (RAPD) [22, 23], simple sequence repeats (SSR) [20, 24, 25], amplified fragment length polymorphism (AFLP) [22], insertion–deletion (InDel) [26], intron length polymorphism (ILP) [9, 27], and single nucleotide polymorphism (SNP) [19, 28]. These studies demonstrate that molecular markers can effectively authenticate tea varieties and clarify their genetic relationships. Among these markers, ILP markers are particularly valuable for tea cultivar classification due to their high abundance, substantial polymorphism, reliability, and cross-species transferability [9, 27], making them suitable for plant genetic diversity analyses. ILP markers have also been effectively used for cultivar identification and germplasm evaluation. Our previous study used ILP markers to analyze the genetic diversity and phylogenetic relationships of 176 terraced *C. tetracocca* samples, revealing moderate genetic diversity in this type [27]. Similarly, studies on wild *C. tetracocca* germplasm resources in Pu’an using ILP markers indicated that wild tea resources possess rich genetic diversity and high utilization value [9], with higher genetic diversity in Qingshan Town than in Xindian Town, suggesting that geographical factors play an important role in shaping the genetic diversity pattern of wild *C. tetracocca*. Qingshan Town is the core distribution area of *C. tetracocca*, yet few reports have focused on the genetic diversity of *C. tetracocca* resources in this region. Therefore, evaluating the *C. tetracocca* resources in this area will facilitate the discovery, innovative utilization, and protection of these precious germplasm resources. In this study, we continued to use ILP markers to analyze the genetic diversity of 192 tea plants from four groups in Qingshan Town: wild ancient tea plant Ⅰ, wild ancient tea plant Ⅱ, cultivated ancient tea plant, and cultivated tea plant. The results of this study aim to provide a theoretical basis for the identification, protection, and utilization of unique tea resources in Pu’an and Guizhou Province, and support future breeding efforts.

## Materials and methods

### Plant materials

*C. tetracocca* Chang is a tea plant species endemic to Pu’an County, Guizhou Province, China. Zhang Hongda (H.T. Chang) assigned this Latin name in 1981 based on the species’ distinctive fruit characteristics. Type specimen: *Typus*, China: Guizhou Province, Pu’an County, Pubai Forest Farm, collected by Guizhou Agricultural Products Procurement Bureau, specimen number 02, deposited in the Herbarium of the Institute of Botany, Chinese Academy of Sciences (PE), Beijing, China [29]. Young leaf samples of current-year, vigorously growing shoots for this study were collected from wild and cultivated populations of *C. tetracocca* in Qingshan Town, Pu’an County, Guizhou Province, China. Formal identification of the plant material was conducted by Wenmin Guo, a local tea plant management specialist with long-term experience in tea plant resource research and management in Pu’an County. Voucher specimens of the plant materials used in this study have been deposited in the College of Landscape Architecture and Horticulture Sciences, Southwest Forestry University, Kunming 650,224, China.

**Fig. 1:**
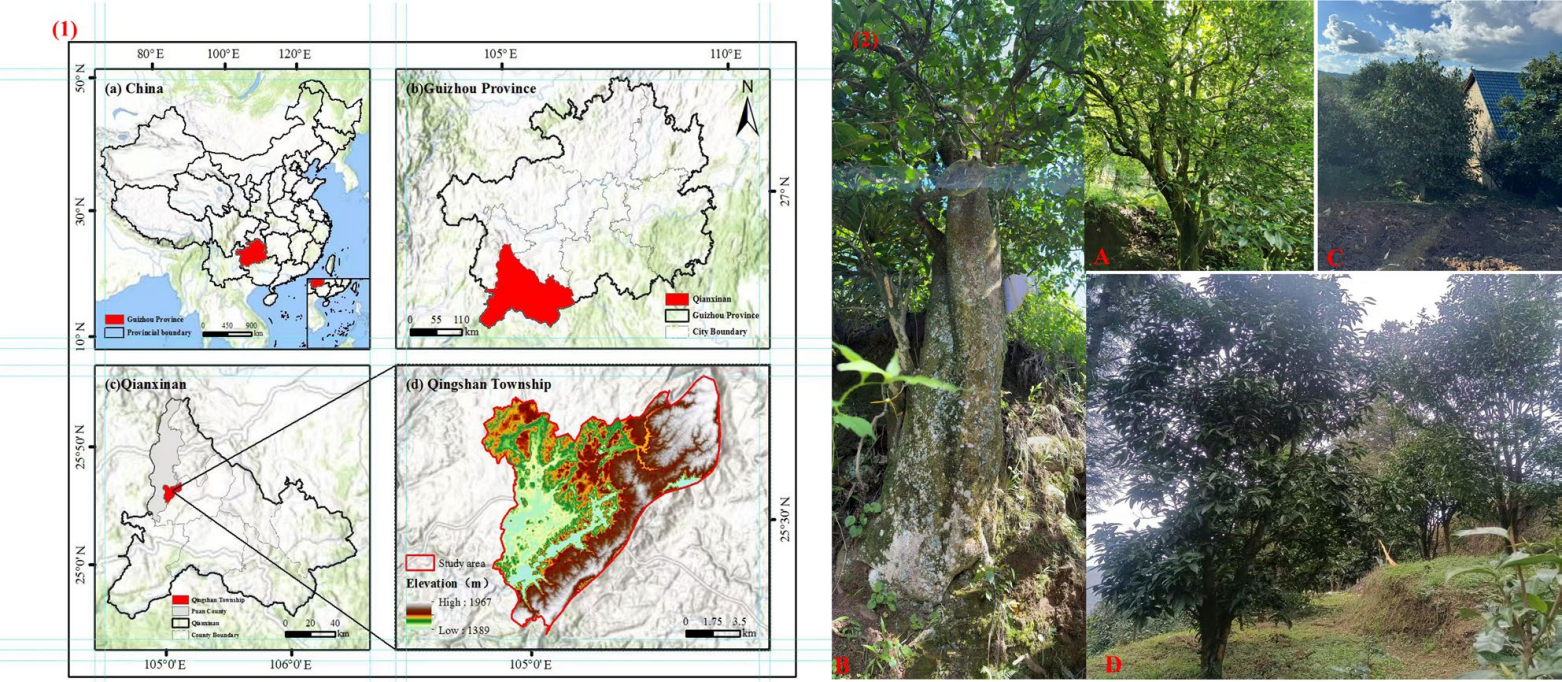
Location map of Qingshan Town (1) and four types of tea plants (2). (1) shows the location of China (**a**), Guizhou Province (**b**), Qianxinan Buyei and Miao Autonomous Prefecture (**c**), and Qingshan Town (**d**) in Guizhou Province. (2) shows representative plants of **A** (wild ancient tea plant Ⅰ), **B** (wild ancient tea plant Ⅱ), **C** (cultivated ancient tea plant), and **D** (cultivated tea plant), respectively

The tea accessions were classified into three categories based on their cultivation status and basal diameter: (1) wild ancient tea plants: individuals naturally growing in the wild with basal diameter exceeding 30 cm; (2) cultivated ancient tea plants: individuals transplanted from mountainous areas by local residents and subjected to over a century of cultivation and domestication; (3) cultivated tea plants: accessions propagated from seeds and cultivated by local residents for 20–40 years. The wild ancient *C. tetracocca* population in Qingshan Town was further divided into two types based on morphological and geographical characteristics. During sampling, individuals in Hama Village were found to have significantly larger basal diameters, and higher distribution elevations compared to those in other areas of Qingshan Town. Consequently, the wild ancient tea plants from Hama Village were designated as a separate type. Specifically, wild ancient *C. tetracocca* from other areas of Qingshan Town (excluding Hama Village) were labeled as Population A (wild ancient tea plant Ⅰ), while those from Hama Village were labeled as Population B (wild ancient tea plant Ⅱ). In total, 192 individuals were sampled across four populations: 72 from Population A, 18 from Population B, 47 from cultivated ancient *C. tetracocca* (Population C), and 55 from cultivated *C. tetracocca* (Population D) (Table 1). After collection, all leaf samples were immediately placed in in silica gel-filled zip-lock bags for rapid desiccation, and detailed labels were affixed to each sample (Fig. 1).

**Table 1:** Information on *C. tetracocca* resources in Qingshan Town

Pop.	Sample size	Longtitude (^°^E)	Latitude (^°^*N*)	Altitude (m)	Basal diameter (cm)	Tea plant information
A	72	104.97-104.98	25.44–25.45	1691–1702	32–64	Labeled by local government as over 100 years old
B	18	104.96-104.97	25.43–25.44	1731–1744	60–96	Labeled by local government as over 1,000 years old
C	47	104.98-104.99	25.43–25.44	1680–1701	35–72	Transplanted by early settlers from mountains to village surroundings, labeled by local government as over 100 years old
D	55	104.96-104.97	25.42–25.43	1697–1721	18–39	Cultivated tea plants aged 20–40 years

Note: A, wild ancient tea plant Ⅰ; B, wild ancient tea plant Ⅱ; C, cultivated ancient tea plants; D, cultivated tea plant

### ILP Primer resources

We amplified genomic DNA from six randomly selected *C. tetracocca* samples using 230 ILP primer pairs, and finally obtained 40 primer pairs with high polymorphism and clear amplified bands for the genetic diversity analysis of the tea germplasm [27]. Detailed information on these 40 primer pairs is provided in Table S1.

### DNA extraction, PCR amplification, and product detection

Genomic DNA was extracted from *C. tetracocca* leaf samples using a modified cetyltrimethylammonium bromide (CTAB) method, following the protocol described by Tang et al. [30]. Polymerase chain reaction (PCR) amplification was performed in a 10 µL reaction mixture containing 100 ng of total genomic DNA, 0.5 µL of each primer (10 µmol/µL), and 7.5 µL of 2× Taq Master Mix [31]. The PCR program was set as follows: an initial denaturation at 94 ℃ for 3 min; followed by 13 cycles of denaturation at 94 ℃ for 30 s, annealing at 65 ℃ for 45 s with a decrement of 0.7 ℃ per cycle, and extension at 72 ℃ for 1 min; followed by another 23 cycles of denaturation at 94 ℃ for 30 s, annealing at 56 ℃ for 45 s, and extension at 72 ℃ for 1 min; and a final extension at 72 ℃ for 5 min. The PCR amplification products were separated by electrophoresis on a 6% non-denaturing polyacrylamide gel.

### Genetic diversity analysis

Clearly visible bands were scored as “1” (present), and the absence of bands was scored as “0”, generating raw binary (0/1) data for subsequent analyses. The binary data were converted into genotypic data using DataFormater software [32]. Genetic diversity parameters, including the number of observed alleles (*N*_a_), effective number of alleles (*N*_e_), Shannon’s information index (*I*), observed heterozygosity (*H*_o_), expected heterozygosity (*H*_e_), Nei’s gene diversity (*H*), and gene flow (*N*_m_), were calculated using Popgene 1.31 software [33]. Allele frequencies derived from this analysis were used to calculate the polymorphic information content (*PIC*) of each primer using the PIC_CALC tool (version 0.6). Genetic diversity parameters among the four populations were compared by nonparametric Kruskal–Wallis test using Origin 24. Genetic variation within and among populations was assessed using the WINAMOVA v1.55 program, and the significance of variance components was evaluated via nonparametric permutation tests with 1000 permutations for statistical validation [34].

### Genetic population structure analysis

The genetic structure of the 192 *C. tetracocca* individuals was analyzed using STRUCTURE 2.3.4 software [35], with the number of hypothetical subpopulations (K) ranging from 2 to 10. For each K value, the analysis was repeated 10 times to ensure reliability, with a burn-in period of 50,000 iterations followed by 100,000 Markov chain Monte Carlo (MCMC) iterations. The optimal K value was determined based on the likelihood value (Ln P(D)) and ΔK statistic [36]. Cluster analysis was performed using MEGA11 (v. 11.0.13) [37] based on genetic distance, employing the Unweighted Pair Group Method with Arithmetic Averages (UPGMA), and a dendrogram was constructed to visualize genetic relationships. Principal Coordinate Analysis (PCoA) was conducted using GenALEx 6.503 software [38] to graphically represent the genetic relationships and variations among individuals and populations.

### Core collection construction and comparison of sampling rates

The optimal core collection size was determined using the R software package Core Hunter 3 (http://www.corehunter.org) [39], based on the genetic distance matrix computed by GenALEx. To identify the optimal sampling rate, the genetic diversity parameters of core collections generated at sampling rates of 15%, 20%, 25%, and 30% were compared with those of the original germplasm collection. Genetic diversity parameters of the whole collection and core collection were compared by nonparametric Kruskal–Wallis test using Origin 24.

## Results

### Genetic diversity analysis of *C. tetracocca* Qingshan Town

Genetic diversity analysis was conducted on 192 *C. tetracocca* individuals collected from Qingshan Town, Pu’an County (Table 2). A total of 180 observed alleles (Na) were detected across 40 ILP loci, with the number of observed alleles per locus ranging from 2 to 10 and a mean of 4.45. The effective number of alleles (*N*_e_) ranged from 1.25 to 8.49, with an average of 2.90. Observed heterozygosity (*H*_o_) varied from 0.00 to 0.93 (mean = 0.22), and expected heterozygosity (*H*_e_) ranged from 0.26 to 0.88 (mean = 0.59). Shannon’s information index (*I*) varied from 0.39 to 2.21 (mean = 1.11), and Nei’s gene diversity index (*H*) ranged from 0.20 to 0.88 (mean = 0.59). The major allele frequency (*MAF*) ranged from 0.17 to 0.89 (mean = 0.51), and the polymorphic information content (*PIC*) varied from 0.18 to 0.85 (mean = 0.53).

**Table 2:** Genetic diversity parameters of 192 *C. tetracocca* samples based on 40 ILP markers

Primer	*N*_a_	*N*_e_	H	I	H_o_	H_e_	PIC	MAF
Tea_ILP1116	6.00	4.09	0.76	1.53	0.00	0.76	0.72	0.32
Tea_ILP1418	4.00	2.35	0.57	0.96	0.93	0.58	0.48	0.47
Tea_ILP1396	4.00	2.58	0.61	1.03	0.30	0.61	0.53	0.48
Tea_ILP1000	5.00	2.96	0.66	1.28	0.00	0.66	0.61	0.22
Tea_ILP1589	3.00	1.77	0.43	0.78	0.29	0.44	0.39	0.73
Tea_ILP900	3.00	1.80	0.45	0.77	0.44	0.45	0.39	0.71
Tea_ILP1097	2.00	1.99	0.50	0.69	0.00	0.50	0.37	0.53
Tea_ILP1023	6.00	3.87	0.74	1.53	0.13	0.74	0.70	0.35
Tea_ILP1222	3.00	2.20	0.55	0.93	0.32	0.55	0.49	0.61
Tea_ILP1192	4.00	1.89	0.47	0.91	0.25	0.47	0.44	0.70
Tea_ILP1073	3.00	1.35	0.26	0.50	0.21	0.26	0.24	0.85
Tea_ILP591	7.00	5.47	0.82	1.81	0.00	0.82	0.79	0.29
Tea_ILP1158	5.00	4.31	0.77	1.51	0.51	0.77	0.73	0.27
Tea_ILP072	3.00	1.86	0.46	0.71	0.66	0.46	0.37	0.66
Tea_ILP015	4.00	3.30	0.70	1.27	0.00	0.70	0.64	0.41
Tea_ILP290	2.00	1.99	0.50	0.69	0.00	0.50	0.37	0.53
Tea_ILP380	6.00	4.57	0.78	1.58	0.54	0.78	0.75	0.26
Tea_ILP450	3.00	2.00	0.50	0.81	0.64	0.50	0.42	0.63
Tea_ILP202	4.00	2.25	0.56	0.96	0.00	0.56	0.49	0.59
Tea_ILP284	4.00	3.05	0.67	1.20	0.00	0.67	0.61	0.42
Tea_ILP1875	8.00	7.25	0.86	2.03	0.00	0.86	0.85	0.21
Tea_ILP1946	4.00	3.22	0.69	1.24	0.00	0.69	0.63	0.41
Tea_ILP1986	10.00	8.49	0.88	2.21	0.00	0.88	0.87	0.17
Tea_ILP2114	7.00	3.77	0.74	1.55	0.00	0.74	0.70	0.39
Tea_ILP2142	4.00	1.92	0.48	0.87	0.03	0.48	0.43	0.68
Tea_ILP2171	5.00	2.98	0.66	1.31	0.00	0.67	0.62	0.51
Tea_ILP1923	3.00	2.11	0.53	0.83	0.77	0.53	0.43	0.57
Tea_ILP1924	6.00	1.83	0.45	0.98	0.00	0.46	0.43	0.72
Tea_ILP1945	4.00	2.19	0.54	0.97	0.58	0.55	0.48	0.60
Tea_ILP1951	4.00	2.04	0.51	0.91	0.39	0.51	0.46	0.66
Tea_ILP1967	4.00	3.07	0.67	1.17	0.00	0.68	0.61	0.36
Tea_ILP1982	5.00	1.70	0.41	0.82	0.00	0.41	0.38	0.75
Tea_ILP1991	4.00	2.27	0.56	1.00	0.58	0.56	0.49	0.59
Tea_ILP2017	6.00	4.10	0.76	1.54	0.00	0.76	0.72	0.36
Tea_ILP2551	3.00	1.61	0.38	0.60	0.47	0.38	0.31	0.75
Tea_ILP3195	5.00	2.63	0.62	1.13	0.00	0.62	0.55	0.46
Tea_ILP1959	3.00	1.98	0.49	0.78	0.68	0.50	0.40	0.62
Tea_ILP3087	3.00	1.25	0.20	0.39	0.20	0.20	0.18	0.89
Tea_ILP1953	4.00	2.94	0.66	1.15	0.00	0.66	0.59	0.42
Tea_ILP2343	5.00	3.11	0.68	1.29	0.00	0.68	0.62	0.41
Mean	4.45	2.90	0.59	1.11	0.22	0.59	0.53	0.51

Note: *N*_a_, number of observed alleles; *N*_e_, effective number of alleles; *H*, Nei’s gene diversity index; *I*, Shannon’s information index; *H*_o_, observed heterozygosity; *H*_e_, expected heterozygosity; *PIC*, polymorphic information content; *MAF*, major allele frequency

Two loci, *Tea_ILP1073* (*PIC* = 0.24) and *Tea_ILP3087* (*PIC* = 0.18), exhibited low polymorphism (*PIC* < 0.25), while the remaining 38 loci showed moderate to high polymorphism (*PIC* ≥ 0.25). Botstein et al. [40] defined loci with a *PIC* > 0.5 as highly polymorphic, those with *PIC* between 0.25 and 0.5 as moderately polymorphic, and those with *PIC* < 0.25 as lowly polymorphic. These results indicate that the 40 ILP primers used in this study displayed a high level of polymorphism across the 192 *C. tetracocca* samples.

Genetic diversity analysis across the four *C. tetracocca* populations (Table 3) showed that *N*_a_ ranged from 3.13 to 4.30, *N*_e_ from 2.27 to 2.99, and *I* from 0.86 to 1.13. Population A (wild ancient tea plant Ⅰ) had the highest *I* value (1.13), whereas Population B (wild ancient tea plant Ⅱ) had the lowest (0.86). Consistently, Nei’s gene diversity (*H*) followed the same pattern, with values of 0.60, 0.50, 0.56, and 0.55 for Populations A, B, C, and D, respectively. Population A also had the highest *H* value (0.60), and Population B the lowest (0.50). The congruent trends of *I* and *H* indices indicated that Population A harbors the most complex genetic background and the highest genetic diversity, whereas Population B shows the lowest genetic diversity among the four populations.

**Table 3:** Genetic diversity analysis of four *C. tetracocca* populations in Qingshan Town

Population	Sample size	*N*_a_	*N*_e_	I	H	H_o_	H_e_	PIC	MAF
A	72	4.30^a^	2.99^a^	1.13^a^	0.60^a^	0.19^a^	0.61^a^	0.55^a^	0.51^b^
B	18	3.13^b^	2.27^b^	0.86^b^	0.50^a^	0.24^a^	0.51^b^	0.45^b^	0.59^a^
C	47	4.03^a^	2.64^ab^	1.02^ab^	0.56^a^	0.25^a^	0.57^ab^	0.50^ab^	0.55^ab^
D	55	4.10^a^	2.57^ab^	1.01^ab^	0.55^a^	0.24^a^	0.55^ab^	0.49^ab^	0.56^ab^

Note: A, wild ancient tea plant Ⅰ; B, wild ancient tea plant Ⅱ; C, cultivated ancient tea plant; D, cultivated tea plant. *N*_a_, number of observed alleles; *N*_e_, effective number of alleles; *H*, Nei’s gene diversity index; *I*, Shannon’s information index; *H*_o_, observed heterozygosity; *H*_e_, expected heterozygosity; *PIC*, polymorphic information content; *MAF*, major allele frequency. Different letters indicate statistically significant differences (*P* < 0.05)

### Genetic similarity and genetic distance of *C. tetracocca* population

Genetic similarity and genetic distance among the four *C. tetracocca* populations were analyzed (Table 4). All populations exhibited high genetic similarity coefficients and correspondingly small genetic distances. Cultivated ancient tea plants (Population C) and cultivated tea plants (Population D) had the highest genetic similarity coefficient (0.95) and the smallest genetic distance (0.06). In contrast, the two wild ancient tea plant populations (A and B) had the lowest genetic similarity coefficient (0.89) and the largest genetic distance (0.12). These results indicate that the cultivated tea populations are genetically most closely related, whereas the two wild ancient tea plant populations show the greatest genetic divergence and the most distant phylogenetic relationship.

**Table 4:** Genetic similarity (upper triangle) and genetic distance (lower triangle) among *C. tetracocca* populations in Qingshan Town

Population	A	B	C	D
A	1.00	0.89	0.93	0.94
B	0.12	1.00	0.92	0.92
C	0.08	0.09	1.00	0.95
D	0.06	0.08	0.06	1.00

Note: A, wild ancient tea plant Ⅰ; B, wild ancient tea plant Ⅱ; C, cultivated ancient tea plant; D, cultivated tea plant

### Genetic differentiation among *C. tetracocca* populations

Genetic differentiation coefficients for the four *C. tetracocca* populations were calculated using Popgene (Table 5). The inbreeding coefficient within populations (*F*_is_) was 0.58, the total inbreeding coefficient (*F*_it_) was 0.60, and the population differentiation coefficient (*F*_st_) was 0.05. This indicates that only 5% of the genetic variation occurred among populations, while 95% occurred within populations, suggesting that the vast majority of genetic variation in *C. tetracocca* in Qingshan Town originates from within populations. The gene flow (*N*_m_) value was 5.22, indicating frequent genetic exchange among the four populations. AMOVA analysis further confirmed this pattern, showing that 4.46% of the genetic variation stemmed from differences among populations and 95.54% from within populations (PhiST = 0.045, *P* < 0.001).

**Table 5:** Genetic differentiation among four *C. tetracocca* populations in Qingshan Town

POPGENE					AMOVA				
*F*_is_	*F*_it_	*F*_st_	*N*_m_		Source of variation	*df*	*SS*	Var. components	*PMV*%
0.58	0.60	0.05	5.22		Among population	3	202.63	1.01	4.46 (PhiST = 0.045, *P* < 0.001)
					Within populations	188	4070.94	21.65	95.54

Note: *F*_is_, inbreeding coefficient within populations; Fit, total inbreeding coefficient; *F*_st_, population differentiation coefficient; *N*_m_, gene flow; *df*, degrees of freedom; *SS*, sum of squares; Var. components, variance components; *PMV*%, percentage of molecular variation; PhiST, Phi-statistic for molecular variance

### Population structure and phylogenetic relationship of *C. tetracocca*

**Fig. 2:**
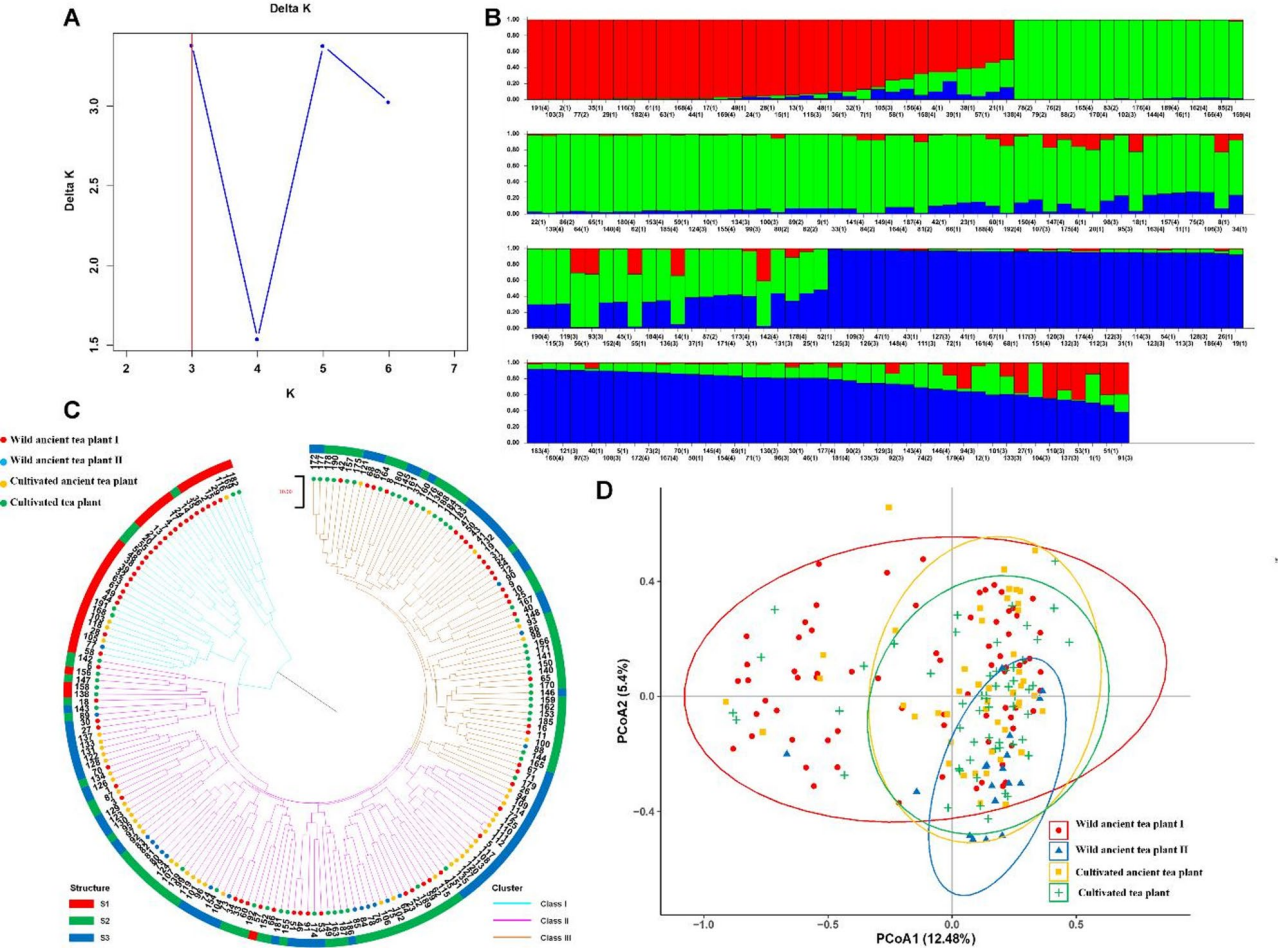
Genetic structure and phylogenetic relationships of 192 *C. tetracocca* individuals. **A **ΔK values calculated from the posterior probability distribution across different K values (2–10). **B **Population structure analysis for K = 3; the 192 accessions are divided into three subpopulations (S1: red, S2: green, S3: blue), with each vertical bar representing an individual and the color proportion indicating the ancestry contribution from each subpopulation. **C **UPGMA phylogenetic tree dividing the 192 individuals into three clusters (Cluster I: blue branch, Cluster II: purple branch, Cluster III: orange branch); the outer ring color codes represent tea plant types (red: wild ancient tea plant Ⅰ, blue: wild ancient tea plant Ⅱ, orange: cultivated ancient tea plant, green: cultivated tea plant). **D **PCoA plot of the four *C. tetracocca* populations (red dots: wild ancient tea plant Ⅰ, blue triangles: wild ancient tea plant Ⅱ, orange squares: cultivated ancient tea plant, green crosses: cultivated tea plant)

Bayesian model-based clustering using STRUCTURE software was performed to analyze the genetic structure of the 192 *C. tetracocca* individuals. The Evanno method (Δ*K*) identified the optimal number of subpopulations (*K*) as three, corresponding to the highest Δ*K* value (Fig. 2A). All individuals displayed admixed ancestry with varying contributions from the three inferred genetic groups, indicating extensive historical gene flow among the populations (Fig. 2B). Individuals with an ancestry coefficient of *Q* > 0.60 were assigned to the corresponding pure genetic groups, whereas those with *Q* ≤ 0.60 were classified as admixed individuals [41–42]. Using this threshold, all 192 individuals were assigned to three distinct subpopulations (S1, S2, and S3). In total, 90.63% of individuals (174 of 192) were assigned to these three genetic subpopulations, while the remaining 9.37% (18 individuals) were identified as admixed genotypes (*Q* ≤ 0.60) (Table 6; Table S2). Of the admixed genotypes, 17 were moderately admixed (0.40 ≤ *Q* ≤ 0.60), and only one individual (0.52%) exhibited weak genetic assignment (*Q* < 0.40) under the defined thresholds.

**Table 6:** Classification of individual genetic components based on STRUCTURE analysis

Subpopulation	Sample size	Q > 0.60	0.40 ≤ Q ≤ 0.60	Q < 0.40
S1	34	32 (94.12%)	2 (5.88%)	0 (0.00%)
S2	87	78 (89.66%)	9 (10.34%)	0 (0.00%)
S3	71	64 (90.14%)	6 (8.45%)	1 (1.41%)
Total	192	174 (90.63%)	17 (8.85%)	1 (0.52%)

The red subpopulation (S1) comprised 34 samples, including 22 from Population A (64.70%), 1 from Population B (2.94%), 4 from Population C (11.76%), and 7 from Population D (20.59%). The green subpopulation (S2) included 87 samples: 27 from Population A (31.03%), 14 from Population B (16.09%), 14 from Population C (16.09%), and 32 from Population D (36.78%). The blue subpopulation (S3) had 71 samples: 23 from Population A (32.39%), 3 from Population B (4.23%), 29 from Population C (40.85%), and 16 from Population D (22.54%). These results indicate no significant genetic differentiation among the four *C. tetracocca* populations in Qingshan Town. Population A was relatively evenly distributed across the three subpopulations (S1: 22/72, S2: 27/72, S3: 23/72); Population B was mainly concentrated in S2 (14/18); Population C was predominantly distributed in S3 (29/47); and Population D was mainly found in S2 (32/55) and S3 (16/55).

A UPGMA phylogenetic tree was constructed to explore the genetic relationships among the 192 *C. tetracocca* individuals (Fig. 2C), which divided the samples into three clusters consistent with the STRUCTURE subpopulation divisions. Cluster I included 34 samples, with 25 from Population A (73.53%), 1 from Population B (2.94%), 4 from Population C (11.76%), and 4 from Population D (11.76%). Cluster II had 92 samples: 23 from Population A (25.00%), 14 from Population B (15.22%), 36 from Population C (39.13%), and 19 from Population D (20.65%). Cluster III comprised 66 samples: 24 from Population A (36.36%), 3 from Population B (4.55%), 7 from Population C (10.61%), and 32 from Population D (48.49%). Cluster I and STRUCTURE subpopulation S1 were both primarily composed of individuals from Population A, while other tea plant types were scattered across different clusters and subpopulations, confirming low genetic differentiation among the four populations.

PCoA analysis showed that the four *C. tetracocca* populations in Qingshan Town exhibited extensive genetic overlap with no clear separate clustering (Fig. 2D). A small number of individuals from Population A were relatively dispersed and slightly separated from other populations in the PCoA plot, which may indicate unique genetic variation within this subset of individuals.

### Core collection construction and sampling rate comparison

A core collection of *C. tetracocca* was constructed using Core Hunter based on the genetic distance matrix calculated by GenALEx v. 6.503. The genetic diversity parameters of core collections generated at sampling rates of 15%, 20%, 25%, and 30% were compared with those of the original collection (Table 7). *I*, *H*_e_, *PIC* of the core collections were higher than those of the original collection, and these indices increased with decreasing sampling rates. In contrast, *N*_a_, *H*_o_, and *MAF* decreased with decreasing sampling rates.

**Table 7:** Genetic diversity of the original *C. tetracocca* germplasm collection and core collections at different sampling rates

Original	Sample size	Na	I	Ho	He	MAF	PIC
	192	4.45^a^	1.11 ^a^	0.22 ^a^	0.59 ^a^	0.51 ^a^	0.53 ^a^
15%	29	4.30^a^	1.24 ^a^	0.16 ^a^	0.67 ^a^	0.44 ^a^	0.61 ^a^
20%	39	4.40^a^	1.24 ^a^	0.16 ^a^	0.66 ^a^	0.45 ^a^	0.60 ^a^
25%	48	4.43^a^	1.23 ^a^	0.17 ^a^	0.65 ^a^	0.46 ^a^	0.59 ^a^
30%	58	4.45^a^	1.22 ^a^	0.17 ^a^	0.65 ^a^	0.46 ^a^	0.60 ^a^

Note: *N*_a_, number of observed alleles; *I*, Shannon’s information index; *H*_o_, observed heterozygosity; *H*_e_, expected heterozygosity; *MAF*, major allele frequency; *PIC*, polymorphic information content. Different letters indicate statistically significant differences between a core subset and the original population (*P* < 0.05)

**Fig. 3:**
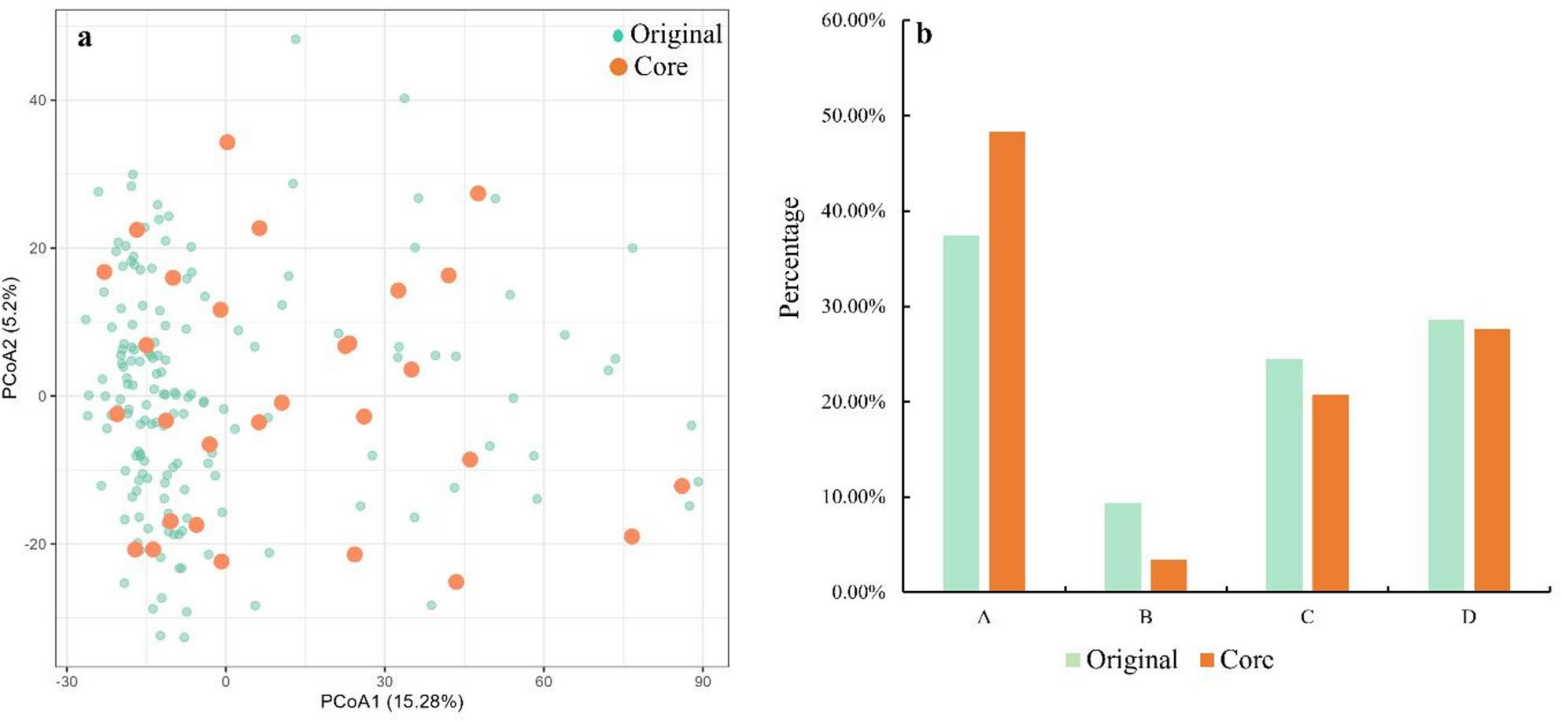
PCoA analysis (**a**) and population proportion comparison (**b**) of the original *C. tetracocca* collection (*n* = 192) and the core collection (*n* = 29). In (**a**), black dots represent the original collection and red dots represent the core collection; in (**b**), different colors represent four tea plant populations (A: wild ancient tea plant Ⅰ, B: wild ancient tea plant Ⅱ, C: cultivated ancient tea plant, D: cultivated tea plant)

PCoA analysis demonstrated that the core collection individuals were evenly dispersed across the entire genetic range of the original collection (Fig. 3a), indicating that the core collection effectively captures the genetic diversity of the original germplasm. Based on the genetic diversity parameters and PCoA results, a 15% sampling rate was identified as optimal, and a core collection comprising 29 accessions was subsequently established (Table S3). This core collection included 14 accessions from Population A, 1 from Population B, 6 from Population C, and 8 from Population D, representing 48.28%, 3.45%, 20.69%, and 27.59% of the core collection, respectively. Compared with the original germplasm, the proportion of Population A in the core collection increased from 37.50% to 48.28%, while the proportions of Population B and C decreased from 9.38% to 3.45% and from 24.48% to 20.69%, respectively; the proportion of Population D remained relatively, with a slight change from 28.65% to 27.59% (Fig. 3b).

## Discussion

Genetic diversity is a key determinant of a species’ environmental adaptability and evolutionary potential, analyses of genetic diversity and phylogenetic relationships provide a robust scientific foundation for the collection, conservation, utilization, and breeding of novel cultivars [8, 20, 43]. In this study, we assessed the genetic diversity of 192 *C. tetracocca* individuals from Qingshan Town using 40 ILP markers, representing a larger sample size than our previous studies of wild ancient *C. tetracocca* (138 accessions) [9] and terraced cultivated *C. tetracocca* (176 accessions) [27] in Pu’an County. The average *PIC* value was 0.53, significantly higher than that of the terraced cultivated population (0.48) [27] but slightly lower than that of the pure wild ancient type (0.58) [9], revealing a clear genetic diversity gradient: wild ancient type > Qingshan Town mixed population > cultivated type [44, 45]. This pattern indicates that the *C. tetracocca* population in Qingshan Town represents an intermediate transitional stage between wild and cultivated forms, maintaining higher genetic variation than cultivated types as a result of less intensive artificial selection [46] and frequent natural gene flow between wild and cultivated populations [47, 48]. Nevertheless, its genetic diversity is lower than that of the pure wild ancient germplasm, which may reflect the cumulative impacts of habitat fragmentation and genetic drift caused by human activities [49, 50]. These results emphasize the significance of the Qingshan Town population as a valuable intermediate breeding resource, which can be exploited to expand the genetic basis of cultivated tea varieties .

The *PIC* value of 0.53 in this study is higher than those reported for tea germplasm from Guizhou Province (0.359) [21] and the Wanzhou tea population (0.37) [51], but lower than those documented for tea germplasm from Anhui Province (0.64) [52], Ruchengbaimao tea (0.70) [20], and Huangshan tea (0.83) [18]. Three primary factors may account for these differences. First, different molecular marker systems were employed across studies: SNP markers by Niu et al. [21] and Liu et al. [53], SSR markers by Liu et al. [20] and Ruan et al. [18], and ILP markers in the present study. *PIC* values cannot be directly compared across marker types owing to inherent differences in mutation rates, genomic coverage, and polymorphism detection efficiency [20]. Second, the examined populations exhibit distinct genetic backgrounds and geographic origins, with unique evolutionary histories, effective population sizes, and selection regimes [54–56]. For instance, the Qingshan *C. tetracocca* population represents an endangered endemic species restricted to Pu’an, Guizhou, and its genetic diversity reflects localized variation, whereas the Huangshan tea population consists of widely distributed germplasm with a large historical effective population size [18]. Third, variation in sampling design, including geographic range, sampling intensity, and representation of core germplasm, may also affect *PIC* estimates [57].

Life-history traits and geographic distribution play pivotal roles in shaping the genetic diversity of plant species. In general, endemic species with restricted distribution ranges tend to exhibit lower genetic diversity compared to widespread species [54], a pattern driven by their smaller population sizes and geographic isolation, which may promote local adaptation to specific habitats [55]. In this study, the wild ancient tea plant Ⅱ population exhibited the lowest genetic diversity (with the lowest values for the number of observed alleles, *N*_a_ = 3.13; expected heterozygosity, *H*_e_ = 0.51; etc.), whereas the wild ancient tea plant Ⅰ population displayed the highest genetic diversity (Na = 4.30, He = 0.61). The cultivated ancient tea plant and cultivated tea plant populations were intermediate between these two extremes, with highly similar levels of genetic diversity. This pattern mainly arises from the combined effects of population size, demographic history, and human activities: the wild ancient tea plant Ⅱ population has an extremely small census size (*n* = 18), reflecting a drastically reduced effective population size that has undergone a severe genetic bottleneck or founder effect. The survival and reproduction of only a limited individuals have resulted in substantial allele loss, enhanced genetic drift, and elevated inbreeding (observed heterozygosity, *H*_o_ = 0.24, being significantly lower than *H*_e_) [58, 59], ultimately leading to the lowest genetic diversity. The relatively higher major allele frequency (*MAF* = 0.59) in this population represents a typical signature of a genetic bottleneck, in which rare alleles are eliminated and the remaining genetic variation becomes more homogeneous [60], further supporting its endangered status. In contrast, the wild ancient tea plant Ⅰ population has a larger effective population size (*n* = 72), which likely sustains more efficient natural gene flow and the accumulation of genetic variation, thereby representing the most diverse genetic reservoir of *C. tetracocca* in Qingshan Town. The cultivated populations reflect the dual impacts of artificial selection [28]: on the one hand, artificial selection reduces the genetic base, resulting in lower genetic diversity relative to wild populations; on the other hand, the mixing of multiple germplasm source in traditional cultivation systems maintains moderate genetic diversity, with cultivated ancient tea plants exhibiting slightly higher diversity than cultivated tea plants owing to long-term historical accumulation. All four populations showed Ho values lower than He values, indicating prevalent inbreeding—this suggests that wild *C. tetracocca* populations are threatened by habitat fragmentation [61], while cultivated populations require protection against genetic homogenization.

Analysis using STRUCTURE software revealed that the optimal number of genetic clusters was three (*K* = 3), indicating that the genetic composition of this germplasm is best described by a model with three ancestral components. All individuals displayed mixed ancestry, with varying contributions from each ancestral component, providing evidence for extensive historical gene flow among populations. When individuals were assigned to three subgroups (S1, S2, S3) based on an ancestry coefficient threshold (*Q* > 0.60), the phenotypic categories—wild ancient tea plant Ⅰ, wild ancient tea plant Ⅱ, cultivated ancient tea plant, and Cultivated tea plant—were not confined to specific subgroups but distributed across all three. The wild ancient tea plant Ⅰ population was relatively evenly distributed among the three subgroups, consistent with its broad genetic base [62]. In contrast, the wild ancient tea plant Ⅱ population was predominantly assigned to S2, the cultivated ancient tea plant population was most frequent in S3, and the cultivated tea plant population occurred mainly in S2 and S3. This overlapping distribution across multiple subgroups indicates weak overall genetic differentiation among populations, while the formation of the three distinct genetic subpopulations— which exhibited a partial but non-strict one-to-one correspondence with the four geographic populations of *C. tetracocca* collected from Qingshan Town, Pu’an County, Guizhou Province—can be primarily attributed to the combined effects of natural geographic isolation and long-term human management. On the one hand, the region’s complex topography and fragmented mountainous habitats likely restrict pollen and seed dispersal, thereby limiting gene flow among populations and facilitating gradual genetic differentiation over time, making natural geographic isolation a major driver of distinct genetic subpopulation formation. On the other hand, long-term artificial domestication, clonal propagation, germplasm translocation, and seedling exchange among farmers have profoundly altered the spatial distribution of genetic variation; these anthropogenic activities have weakened the association between genetic background and sampling location, leading to the sharing of genetic components across populations.

Phylogenetic analysis using UPGMA grouped the samples into three major clades, which aligned with the STRUCTURE subgroup divisions: notably, the first clade and subgroup S1 were both primarily composed of wild ancient tea plant Ⅰ individuals, whereas other types were scattered across clades and subgroups. This pattern suggests a lack of strict reproductive isolation or deep genetic divergence, and points to ongoing gene flow among groups. PCoA further showed that although the four tea types formed somewhat separate clusters, there was considerable overlap among them, visually underscoring genetic continuity and admixture. Interestingly, within the wild ancient tea plant populations, several individuals from subgroup S1 appeared relatively dispersed in the PCoA plot and were somewhat separated from other populations. This may indicate unique genetic variation within this subset or reflect more complex historical population dynamics.

Constructing a core germplasm bank is critical for the efficient management and conservation of germplasm resources, especially for perennial self-incompatible plant species [52, 63]. *C. tetracocca* is a unique endemic species to Pu’an, Guizhou, and its wild populations are recognized as an endangered protected taxon, with its core distribution area located in Qingshan Town. Accordingly, establishing a core germplasm collection of *C. tetracocca* from Qingshan Town is great significance for optimizing the conservation and utilization of tea germplasm resources. In the present study, a core germplasm bank comprising of 29 *C. tetracocca* individuals was successfully constructed, representing 15% of the original 192 accessions (Table 6). Notably, 14 accessions belonging to wild ancient tea plant Ⅰ population were preserved in the core collection, accounting for 48.28% of the total core germplasm (Fig. 3b). Wild ancient tea plant Ⅰ population exhibited higher genetic diversity than wild ancient tea plant Ⅱ population and cultivated ancient tea plant population (Table 3), which explains its relatively high representation in the core germplasm bank. These results emphasize the key role of wild ancient tea plant Ⅰ population in germplasm resource conservation.

In response to the genetic distribution characteristics of *C. tetracocca* resources in Qingshan Town—where wild ancient tea plant Ⅱ population is scarce and exhibit the lowest genetic diversity, the wild ancient tea plant Ⅰ population is abundant with the highest genetic diversity, and the genetic diversity of cultivated ancient tea plants and cultivated tea plants is slightly lower than that of the wild ancient tea plant Ⅰ population—it is recommended to adopt an integrated strategy combining graded protection with scientific utilization [63]. First, the wild ancient tea plant Ⅰ *C. tetracocca* population, which possesses the highest genetic diversity, should be designated as the core conservation targets [64]. In-situ conservation sites should be established within their concentrated distribution areas to ensure that natural succession and gene flow processes remain undisturbed. Concurrently, priority should be given to germplasm collection from these populations to preserve their abundant alleles, providing a foundation for subsequent breeding efforts. Second, for wild ancient tea plant Ⅱpopulation of *C. tetracocca*—which is scarce in number, exhibit the lowest genetic diversity, but may carry ancient and unique genes—strict ex-situ conservation measures should be implemented [65]. This includes individual plant registration and tagging for rescue protection, as well as establishing germplasm preservation nurseries through asexual propagation to prevent the extinction of these genetic resources. Third, regarding cultivated ancient tea plants and cultivated tea plants with slightly lower genetic diversity, systematic investigation and evaluation should be conducted to select elite individuals with special agronomic traits for categorized preservation [52]. Under the premise of protection, moderate development of differentiated products and promotion of ecological planting models can be pursued to enhance their economic value. Finally, a long-term dynamic monitoring system for genetic diversity should be established to conduct in-depth research on the evolutionary relationships among the three genetic groups and the mechanisms underlying their genetic differences. This will provide a scientific basis for continuously optimizing conservation strategies and promoting germplasm innovation centered on the wild ancient tea plant Ⅰ population, thereby achieving the sustainable inheritance and efficient utilization of Qingshan Town’s *C. tetracocca* resources.

In this study, ILP markers were used as the sole molecular tool to evaluate the genetic diversity and population structure of ancient tea germplasm. ILP markers, which are based on length variation in intronic regions, exhibit high stability, codominant inheritance, and simple experimental operation [9, 27], rendering them suitable for preliminary assessments of genetic variation in plant germplasm. Nevertheless, because ILP markers target only intronic regions, their genome coverage is relatively restricted, which may limit their ability to resolve fine‑scale genetic differentiation and population structure, especially in long‑term domesticated and genetically complex ancient tea populations. By comparison, SNPs and SSRs provide substantially higher analytical power. SNPs are characterized by exceptionally high genome‑wide density and coverage, enabling unbiased scanning of the entire genome and reliable detection of subtle genetic divergence, demographic history, and selection signatures [19, 28]. SSRs, by contrast, are distinguished by extremely high polymorphism and PIC, making them particularly powerful for cross‑population genetic comparisons and diversity assessments [24, 25]. Future investigations of ancient tea germplasm should therefore integrate genome‑wide SNP data from resequencing or SNP arrays, combined with polymorphic SSR markers, to achieve more comprehensive profiling of genetic diversity, more robust inference of population structure, and deeper dissection of the genetic basis of adaptation and domestication. Furthermore, the germplasm analyzed in this study was sampled from a relatively narrow geographic range. Expanding sampling to include populations across diverse elevations, ecological habitats, and historical cultivation regions would improve understanding of the broader patterns of genetic diversity in ancient tea resources. Integrating high‑throughput genotypic data with spatial, environmental, and cultivation history data will help clarify the relative roles of natural processes and human activities in shaping the current genetic structure of ancient tea populations.

## Conclusion

In this study, we comprehensively analyzed the genetic diversity of 192 *C. tetracocca* accessions (including wild ancient tea plant Ⅰ, wild ancient tea plant Ⅱ, cultivated ancient tea plant, and cultivated tea plant) from Qingshan Town, Pu’an County, Guizhou Province. Among these four populations, the wild ancient tea plant Ⅰ population exhibited the highest genetic diversity, while the wild ancient tea plant Ⅱ population had the lowest. STRUCTURE, UPGMA, and PCoA analyses revealed that the 192 accessions were divided into three overlapping genetic clusters with no significant population differentiation. Furthermore, a core germplasm set comprising 29 tea samples (15% of the original collection) was successfully established, which effectively captures the genetic diversity of the original germplasm and prioritizes the conservation of wild ancient tea plant Ⅰ. Our findings provide a novel perspective for the genetic improvement and sustainable utilization of tea varieties in this region, and offer important scientific information for breeders to protect and utilize the genetic diversity of this endangered endemic tea species, supporting the development of graded conservation and scientific utilization strategies.

## Supplementary Information

Supplementary Material 1.

## Data Availability

The original contributions presented in the study are included in the article/supplementary material, further inquiries can be directed to the corresponding authors.

## Abbreviations

CTAB: Cetyltrimethylammonium Bromide
PCR: Polymerase Chain Reaction
ILP: Intron Length Polymorphism
N_a_: Number of Observed Alleles
N_e_: Effective Number of Alleles
I: Shannon’s Information Index
H_o_: Observed Heterozygosity
H_e_: Expected Heterozygosity
H: Nei’s Gene Diversity Index
N_m_: Gene Flow
MAF: Major Allele Frequency
PIC: Polymorphic Information Content
F_is_: Inbreeding Coefficient Within Populations
F_it_: Total Inbreeding Coefficient
F_st_: Population Differentiation Coefficient
AMOVA: Analysis of Molecular Variance
MCMC: Markov Chain Monte Carlo
UPGMA: Unweighted Pair Group Method with Arithmetic Averages
PCoA: Principal Coordinate Analysis
